# What I know, what I want to know, what I learned: Activating EFL college students' cognitive, behavioral, and emotional engagement through structured feedback in an online environment

**DOI:** 10.3389/fpsyg.2022.1083673

**Published:** 2023-01-04

**Authors:** Liqaa Habeb Al-Obaydi, Farzaneh Shakki, Ragad M. Tawafak, Marcel Pikhart, Raed Latif Ugla

**Affiliations:** ^1^English Department, College of Education for Human Sciences, University of Diyala, Diyala, Iraq; ^2^Department of English Language and Literature, Faculty of Humanities and Social Sciences, Golestan University, Gorgan, Iran; ^3^Department of Information Technology, Al Buraimi University College, Al Buraimi, Oman; ^4^Faculty of Informatics and Management, University of Hradec Kralove, Hradec Kralove, Czechia; ^5^Department of Islamic Studies in English, Al-Imam Al-Adhdham University College, Baghdad, Iraq

**Keywords:** cognitive engagement, behavioral engagement, emotional engagement, structured feedback, online education

## Abstract

Given the spread of the COVID-19 pandemic, online classes have received special attention worldwide. Since teachers have a lasting effect on the students, the teacher–student relationship is a pivotal factor in language learning classes. Students will not be engaged in class activities if they are not sufficiently challenged by them or if they do not find them interesting, especially in online classes. From this point of view, motivating, engaging, and testing techniques in online classes are highly important. The present study attempts to demonstrate a correlation between structured feedback and three types of engagement in an online class: cognitive, behavioral, and emotional engagement. The structured feedback, which is used at the end of each lesson lets the students express what they know, what they want to know, and what they learned. The sample of the study consists of 114 EFL third-year college students. The study's findings reveal positive and significant correlations between the three types of engagement; cognitive, behavioral, and emotional, and the use of structured feedback in online classes. In a nutshell, some academic implications and recommendations are provided.

## Introduction

Technology has brought inevitable effects on different aspects of human life, particularly education (Aghaei et al., 2020; Derakhshan and Malmir, 2021). Due to the advancement of technology, there has been a shift from traditional classes to online learning during the last decade. How dynamic the students are in online courses can be a simple definition of engagement. Generally, the exploitation of resources (time and effort) by students or instructors to improve the learning experience and the learning results is referred to as engagement (Trowler, 2010). Academic engagement among students is a prerequisite for L2 learning (Dotterer and Lowe, 2011; Nejati et al., 2014; Derakhshan, 2021, 2022c; Shakki, 2022). It is related to “the quality of how students connect or involve themselves in educational activities” (Skinner et al., 2009, p. 495). According to Amerstorfer and Freiin von Münster-Kistner (2021), students' academic engagement depends on many factors related to the learner, teacher, teaching methods, colleagues, and some features in the learning environment.

As stated by many studies such as Carini et al. (2006), Trowler (2010), Wang J. et al. (2022), and Wang et al. (2022a,b), students' satisfaction, persistence, and academic achievement influence the students' engagement, which is where individual's attention is allocated in active reaction to the environment; it is then a growth-producing activity (Csikszentmihalyi, 1990). School engagement (student involvement) has emerged as a critical notion linked to a variety of educational outcomes, such as achievement, behavior, attendance, conduct, and dropout/completion (Jimerson et al., 2003, 2009). Fredricks et al. (2016) stated that there are three components of engagement, which are cognitive, behavioral, and emotional and they are all related to each other in the process of engagement (Wang et al., 2016).

Nowadays, engagement in online teaching and learning has become crucial, especially after the COVID-19 pandemic. This engagement should be characterized by the effort, concentration, active participation, and emotional responsiveness (Philp and Duchesne, 2016; Aghaei et al., 2022). Because of the widespread use of online learning, it is more vital than ever to figure out how engagement relates to students' performance (i.e., the measurement of results) and specific characteristics of the online classroom (i.e., the types of educational practices). Every responsible English language teacher makes every effort to meet the needs of their students to improve their level of performance (Derakhshan and Shakki, 2020). To accomplish this, they employ a variety of approaches and strategies, and all of this can be accomplished effectively if some responses are received from students in the form of feedback. The use of feedback in EFL classes of all types can promote an effective interactive class environment.

This viewpoint was highlighted by Hyland (2006) who mentioned that one of the most crucial jobs of a teacher is to provide feedback to students, as it allows for the kind of individual attention that is otherwise difficult to achieve in a classroom setting. As a kind of feedback, reinforcement and interpersonal attraction theories may also be related to students' engagement (Derakhshan et al., 2019). This theory proposes that once an individual finds anything satisfying in interacting with another person, they will want to communicate with that person again. In educational settings, teachers' non-verbal acts in interacting with their pupils, if deemed gratifying, may contribute to improving student classroom engagement (Witt et al., 2004). As a result, the more regular and constructive the feedback is in online education, the greater the potential for performance improvement (Walther and Burgoon, 1992; Flahery and Pearce, 1998; Eslami and Derakhshan, 2020). In relation to that, the use of structured feedback is highlighted in the present study.

Recently, and within the current wave of online teaching in colleges and schools, the challenge of motivating students to engage in online classes has become urgent. However, if the right pedagogy is not used, the haste to add online classes to the calendar might result in losing connectivity with the students. As mentioned by Shu-Fang and Aust (2008), online learning possesses two distinguished pedagogical features that were inefficient in the earlier generations of distance education. One is interaction and the other is collaboration.

The nature of human–computer interaction (HCI) has changed dramatically in recent decades, transitioning from simple user interfaces to interactive and engaging experiences (Shankar et al., 2016). This study tries to focus on using structured feedback from the students at the end of each online session. Structured feedback as mentioned by (Larsen-Freeman and Anderson, 2011, p. 67) happened when “the students are invited to make observations about the day's lesson and what they have learned. The teacher accepts the students' comments in a non-defensive manner, hearing things that will help give him direction for where he should work when the class meets again.” In the present study, the teacher asked the students to determine what they already know about the material of the lesson (their previous knowledge), what they want specifically to know, and what they actually learned in the class. This technique is used to represent structured feedback in online classes. Thus, this study shows which type of engagement in the students may be developed (cognitive, behavioral, and emotional) and which can be correlated with structured feedback in online classes. This desideratum can be filled out by answering the following research questions:

- Is there a correlation between EFL college students' behavioral engagement and their attitudes toward structured feedback in online classes?
- Is there a correlation between EFL college students' emotional engagement and their attitudes toward structured feedback in online classes?

## Literature review

### Engagement

Engagement has become a popular psychological term that influences human behavior and decision-making in a variety of areas, including education, employment, leisure, and marketing. Kuh (2009) explained students' engagement as “…the more students study a subject, the more they know about it, and the more students practice and get feedback from faculty and staff members on their writing and collaborative problem solving, the deeper they come to understand what they are learning” (p. 5). According to the student involvement theory, the more involved a student is in college, the more learning and personal growth they will receive (Astin, 1984; Wang et al., 2021).

In the literature, most of the previous studies focused on traditional students' engagement in universities worldwide (Robinson and Hullinger, 2008). After the COVID-19 pandemic, all universities worldwide moved into online learning. This movement was unusual to most students, especially for those who have not joined this system yet though most studies proved its positive role in many aspects in relation to language teaching. Because of its virtues and the benefits for English students, researchers consider EFL online classes an innovative approach that can answer students' concerns in English lessons (Tawafak et al., 2019; Alahmadi and Alraddadi, 2020; Hamouda, 2020; Pikhart and Klimova, 2020).

### Empirical studies

In the same concern, Rad et al. (2022) recommend using flipped learning as a way of online education, as it positively impacts instructors' support, team support, and positive subjective feelings about the course material. Moreover, Çakmak et al. (2021) reported the essential role of online education, specifically in vocabulary learning and retention. On the contrary, Pikhart et al. (2022) found some dissatisfaction among students with online education in the EFL context as they much prefer traditional face-to-face classes and written textbooks.

As far as students' engagement is concerned, it could be divided into three interrelated components, which are cognitive, behavioral, and emotional engagement (Fredricks et al., 2016; Al-Bahadli, 2020). Through cognitive engagement, students apply mental energy during the learning process. First, according to Nguyen et al. (2016), cognitive engagements deal with the student's enrolment in the learning process, which refers to the students' improvement in understanding, studying, and getting the knowledge shown in their academic work. This means that cognitive engagement is very important in identifying the students' psychological motivations, which are connected directly with their engagement. Second, in behavioral engagement, the students perform special behaviors while they are learning. According to Nguyen et al. (2016), behavioral engagement is related to students' participation and activities in the classroom that motivates the students to be a part of the school learning environment. Third, while they are learning, students should experience positive emotions to get emotional engagement (Derakhshan, 2022a).

The student's engagement could be considered as the analysis of their positive behaviors, such as students' participation, attendance, and attention (Derakhshan, 2022b). This engagement analyzes the students' psychological experience and their feelings in the schools. Through the chain mediation of autonomous motivation and positive academic emotions (such as satisfaction and relief), teacher engagement had an impact on students' English achievement (Derakhshan et al., 2022; Wang et al., 2022a,b; Wang J. et al., 2022). Another dimension, which is social engagement was also added by Fredricks et al. (2016). This is to recognize that learning possibilities are embedded in a social environment (Wang and Hofkens, 2019), as evidenced by students' participation in social contact or collaboration during the learning process. In this concern, Latipah et al. (2020) demonstrated that students were positively engaged in terms of behavioral, emotional, and cognitive engagement, according to the findings of their study. In terms of behavioral engagement, students made a significant effort to study English before class by watching a video and performing admirably. They are more engaged in learning activities and most students respond positively to emotional engagement as they were enthusiastic about learning English.

Pilotti et al. (2017) reported that the richness of the discussion prompts in classes was found to have a favorable relationship with students' cognitive engagement and instructors' behavioral engagement. With increased class size, both cognitive and behavioral measures of student involvement decreased (Derakhshan and Shakki, 2019). So, the type of engagement varies from context to context depending on the type of techniques used by the teachers and the class environment. Based on that, this study tries to discover the type of correlations that can be existed between the three types of engagement and structured feedback, which is used by teachers in online English classes.

## Methods

### Participants

The total number of participants was 114. They were all EFL third-year college students at the English department of the University of Diyala, Iraq, who were exposed to the structured feedback in their daily lessons for 8 weeks. The ages of the participants varied between 18 and 26 years, but most of them were 18–21 years as shown in Table 1.

**Table 1:** Demographic information.

**Age**	**Participants**	**Percentage**
18–21	75	65.8%
22–26	31	27.2%
Above 26	8	7%
Total	114	100%

According to Table 1, the highest number of participants was from the age of 18–21 years (65.8%), and the less participants were with the age older than 26 years (7%). Regarding the specialization, all 114 students belong to English Department, and they were all third-year students.

### Instruments

The instrument used to collect data, to verify/falsify the hypotheses, was an online questionnaire that the researchers constructed (see Appendix 1). SESQ Students' Engagement in Schools Questionnaire was used, which was constructed by many researchers (see Lam and Jimerson, 2008). The SESQ consisted of 109 items focused on the comprehensive assessment of the construct of students' engagement. The researchers summarized the number of items to suit the study's aim and context. Students' attitudes questionnaire was adopted from a questionnaire developed by Barmby et al. (2008) in the same process of writing the questionnaire (see Lam and Jimerson, 2008). The questionnaire, after a short introduction with the consent to take part in the survey, contained a few demographic questions related to age and specialization. The validity and reliability of the items were confirmed statistically, as shown in **Table 4**. The results show that the sample can be accepted, and the test is statistically valid. Face validity was also gained by exposing the instruments and the idea of the study to some specialists in the domain of the English language and taking their notes into consideration.

In each lesson, the students were invited to register what they noticed in the lecture in three domains; what they already knew about the material of the lecture (their previous knowledge), what they want specifically to know, and what they actually learned in the same lecture. The teacher then, at the end of each lecture, tries to listen to the students, kindly discussing some points, and attempting to respond to all their inquiries. The students were third-year college students and the material is a method of English language teaching, a book by Larsen-Freeman and Anderson (2011) “*Techniques and Principles in Language Teaching.”* The questionnaire was submitted to the participants online *via* Google Forms. The data collection took place in January and February 2022, just at the beginning of the second semester. Once the questionnaire was finalized, the next step would be to test the reliability of the questionnaire using the Cronbach alpha test. If the reliability passed Cronbach's alpha of >0.7, the questionnaire would be distributed to the sample of the study. However, it is always advantageous to pilot the questionnaire first. This is in line with Sekaran and Bougie (2016)'s recommendation. They suggested that before collecting data, useful statistics from the original study should be calculated to ascertain reliability. This section discusses how the acceptance model was piloted in this study.

The pilot test was conducted by one of the researchers on her section's students. They were 58 undergraduate students from two sections. A copy of the questionnaire was distributed during class time. The aim was to check if students could answer the questionnaire without any difficulty. The participants that were selected for the pilot study received a preliminary declaration stating that their participation was voluntary and that their anonymity would be guaranteed if they chose to complete the questionnaire survey.

As shown in Table 2, the internal consistency of the items was measured using Cronbach's alpha analysis on Statistical Package for the Social Sciences (SPSS). Since Cronbach's alpha fell within the acceptable range (0.729–0.916) >0.7, the reliability of the scale was confirmed (Tawafak et al., 2018). This shows that the current model is applicable to the acceptance model and the measures reflect the research goal. The questionnaire was then distributed online using Google Forms and a free online survey service that can be used to collect responses. In the first step, the researchers tested the initial results after the 114 responses received to check whether the survey is working properly or not.

**Table 2:** Reliability indices.

**Latent factors**	**Items (*N*)**	**Cronbach's alpha**
Attitude toward structure feedback	11	0.916
Behavioral engagement	11	0.729
Cognitive engagement	12	0.862
Emotional engagement	8	0.802

### Reliability

Table 3 shows a high acceptance of reliability. The normal acceptance needs to be >0.7, and the current test of this survey gave 0.936 as a significant accepted result.

**Table 3:** Reliability statistics.

**Cronbach's alpha**	**Items (*N*)**
0.936	42

### Conceptual research model

**Figure 1:**
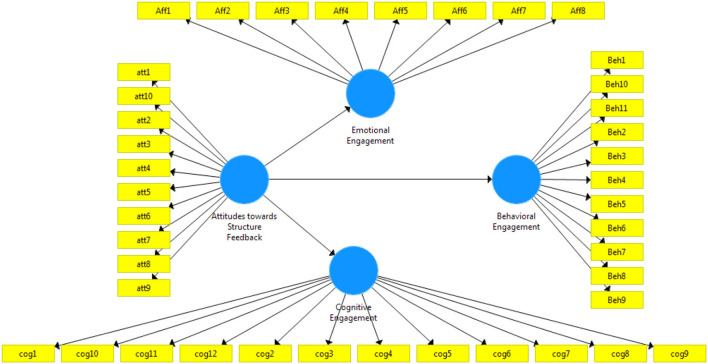
Research model.

The function of construct validity is to validate the assessment that ensures the factors measure what it intends to measure (Mohajan, 2017). This study includes subsections of construct validity, such as the evaluation of reliability and convergent validity, as well as data screening and measurement model. Moreover, the validation of the structural model and hypothesis testing are also described. The survey, which comprised 42 questions, distributed four factors, as shown in Figure 1.

The research paper developed a model consisting of four factors, as shown in Figure 1. Attitude toward structured feedback connected with 11 items of contrasts. Emotional engagement is linked with eight items of the survey questionnaire. Behavioral engagement connected with 11 items, and finally, cognitive engagement used 12-item questions. The main influences individually linked the attitude toward structured feedback with the other three factors.

## Results

Once the acceptance model passed the reliability test, data could be collected. It is important to collect information from every single individual in the population. Hence, sampling means collecting sufficient information from particular participants in the population to popularize the findings of the entire population (Hair et al., 2014). The data to validate the model were collected from four different HEIs from the sample of students. All these four HEIs apply to an online learning system. The next main criterion, therefore, was that the research sites must be using e-learning systems. According to Cone and Foster (1993), a few departments in universities were already using e-learning or had participated in earlier research as the teachers were allowed to use e-learning in combination with their subject knowledge at that point in time.

**Figure 2:**
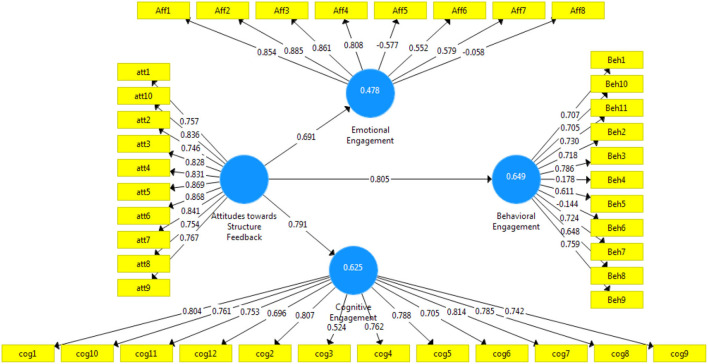
Initial research model results.

Others were still in the early stages of the innovation-decision process or were transferring from a period of investigation into a phase where e-learning was considered part of the institutional agenda (Tawafak et al., 2018). The data entered in an Excel file and saved as vs. extension were tested using PLS-SEM software based on a set of data collection used to evaluate all the questions with different factors. The total number of respondents was 114, making it a representative sample. For this research, the students were considered as the key participants to evaluate the factors and the acceptance of the conceptual research model as mentioned in Figure 2 shows the results of using PLS-SEM construction and the validity of its items and influence links.

As shown in Figure 2, some of the items in the model showed low loadings on their pertinent factors. These items endanger the internal validity of the model and have to be excluded. Table 4 shows the loading for each item used in the model and which questions are the most highly impacted and strongly connected.

**Table 4:** Item loading and reliability.

**Construct**	**Item**	**Loading**	**Remarks**	**Alpha**	**Correlation**
Attitude toward structure feedback	ATT1	0.757		0.878	Supported
	ATT2	0.746	Supported		
	ATT3	0.828	Supported		
	ATT4	0.831	Supported		
	ATT5	0.869	Supported		
	ATT6	0.868	Supported		
	ATT7	0.841	Supported		
	ATT8	0.754	Supported		
	ATT9	0.767	Supported		
	ATT10	0.836	Supported		
Behavioral engagement	Beh1	0.711	S	0.871	Supported
	Beh2	0.721	Supported		
	Beh3	0.785	Supported		
	Beh4	0.178	Not supported		
	Beh5	0.600	Supported		
	Beh6	−0.144	Not supported		
	Beh7	0.726	Supported		
	Beh8	0.652	Supported		
	Beh9	0.760	Supported		
	Beh10	0.712	Supported		
	Beh11	0.729	Supported		
Cognitive engagement	Cog1	0.804		0.927	Supported
	Cog2	0.807	Supported		
	Cog3	0.524	Not supported		
	Cog4	0.762	Supported		
	Cog5	0.788	Supported		
	Cog6	0.705	Supported		
	Cog7	0.814	Supported		
	Cog8	0.785	Supported		
	Cog9	0.742	Supported		
	Cog10	0.761	Supported		
	Cog11	0.753	Supported		
	Cog12	0.696	Supported		
Emotional engagement	Aff1	0.849	Su	0.827	Supported
	Aff2	0.880	Supported		
	Aff3	0.861	Supported		
	Aff4	0.808	Supported		
	Aff5	−0.577	Not supported		
	Aff6	0.552	Not supported		
	Aff7	0.579	Not supported		
	Aff8	−0.058	Not supported		

Table 4 shows the item loading and Cronbach's alpha values for all constructs/factors in the measurement model, which exceeded the recommended threshold values. In summary, the adequacy of the measurement model indicated that all items were reliable indicators of the hypothesized constructs. According to Table 4 and concerning the results of the factor “attitude toward structure feedback,” the highest impact items used are item numbers 3 to 6 and 10, with 0.828, 0.831, 0.869, 0.868, 0.841, and 0.836, respectively. In the behavioral engagement factor, all 11 items are significant and remarks as supported except for items Beh4 (its loading 0.178) and Beh6 (its loading −0.144), and its remarked as not supported by the total questions designed in the survey. In the cognitive engagement factor, all 12 items are remarked as supported items except for Cog3, its item loading value of 0.524, which is <0.6, and remarks as not supported in the survey factors. Although it should be mentioned that Hair et al. (2006) explained that an item with loading above 0.5 can also be supported if the total AVE of the construct is not endangered.

**Figure 3:**
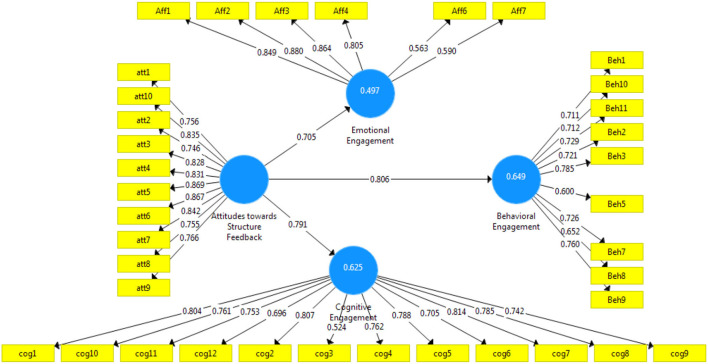
Final research model results.

Regarding the affective section of the survey (emotional engagement) factor in the model design, this factor is supported by all calculations. Moreover, the item loading values are divided into two categories, the first four items are highly supported remarks, while the second four items (items 5 to 8) are not supported because their negative loading values impact the model design. The two of these items (items 6 and 7) had loadings above 0.5 and could be included in the model if they do not endanger the total AVE of the factor. Also, the student feedback was not supporting the results in these last four items. By the end of all PLS calculations, the emotional engagement is remarked as supported and significant results. Based on the results explained earlier, the final model was run after the exclusion of problematic items (Beh4, Beh6, Aff5, and Aff7). The final model is shown in Figure 3.

The path coefficient results for three dependent factors show an acceptable value where all results are above 0.5, the standard condition for being accepted (Table 5).

**Table 5:** Path coefficients.

	**Behavioral engagement**	**Cognitive engagement**	**Emotional engagement**
Attitude toward structure feedback	0.806	0.791	0.705

Table 6 shows the validity of R square results. According to Hair et al. (2006), the R square have three categories: 0 to 0.29 is a weak and mostly rejected model, 0.3–0.45 is acceptable, and from 0.46 to 0.99 is highly accepted and a significant model result. Regarding this analysis, this model is fully accepted with its three R square results constructed directly from the attitude toward structured feedback to the other three related factors with R square 0.649, 0.625, and 0.478 to behavioral engagement, cognitive engagement, and emotional engagement, respectively. Concerning the prediction values of Q square, the accepted results can be any value greater than 0.4. For Table 5, all Q square predicted values are 0.631, 0.613, and 0.434, respectively, which indicates a significant value with all factors.

**Table 6:** *R* square.

	***R* square**	***R* square adjusted**	***Q*^2^-predict**
Behavioral engagement	0.649	0.646	0.629
Cognitive engagement	0.625	0.621	0.626
Emotional engagement	0.497	0.492	0.556

The structural model's characteristics are measured by studying *R* square determination coefficients, regression estimates, and statistical significance. The *R*^2^ value assesses the amount of predictive power and shows the extent of divergence, justified by its antecedent factors in the model. The model's *R*^2^ values should be high enough to reach a minimum level of explanatory power (Urbach and Ahlemann, 2010). Accordingly, *R*^2^ values of 0.67 are considered significant, 0.33 to be reasonable, and 0.19 to be poor. Another measure that is carried out in the assessment of the structural model is the path coefficient value, which measures how strong the link is between the independent factors and dependent factors. To assess if the path coefficient is significant, the value should be higher than 0.100 within the model and be substantive at the 0.05 level of significance at least. Figure 3 shows the real numbers of contrast among factors and their items.

In addition, it shows the active results of *R* square as it is shown in Table 6. In addition to the path coefficient with smooth relationships from the attitude toward structured feedback to the other three factors of the conceptual model as the same values mentioned in Table 5. The standard path coefficient to be accepted should be above 0.5 to prove the link between the factors in the model design.

The convergent validity assesses to what extent the construct measures are different from the other constructs in the model. The value of the convergent validity measure is based on a merge or percentage of variance. Several techniques are employed to measure the relative quantum of convergent validity among measured items. Accordingly, Hair et al. (2006) suggested that the use of factor loadings, composite reliability, and average variance extracted (AVE) in measuring the convergent validity, where factor loadings ≥0.5 and preferably ≥0.70, show a high convergent validity. Moreover, composite reliability with estimates ≥0.70 shows enough convergence or internal consistency. The AVE exhibits the indicator total variance accounted for by the latent construct and the value of the AVEs should be ≥0.5. Thus, when the values are higher than the minimum recommended score for factor loading, composite reliability, and AVE, it signifies the instrument items are valid and reliable.

As can be seen in Table 7, a discriminant validity measure is another test carried out to measure the extent to which a construct is truly different from other constructs. A discriminant validity measure is another test carried out to measure the extent to which a construct is truly different from other constructs. A high discriminating validity shows that a concept is specific and highlights some effects overlooked by other measures. To assess discriminating validity, latent construct correlation matrices are applied where the square roots of the AVEs along with the diagonals are indicated. Correlational statistics between constructs are shown in the lower left off-diagonal elements in the matrix. Thus, discriminant validity is realized when the diagonal elements (square roots of AVEs) exceed the off-diagonal elements (correlations between constructs) in the same row and column as suggested by Fornell and Larcker (1981). Table 8 shows the discriminant validity results.

**Table 7:** Construct reliability and validity.

**Matrix**	**Cronbach's alpha**	**rho_A**	**Composite reliability**	**Average variance extracted (AVE)**
Attitude toward structure feedback	0.878	0.886	0.902	0.657
Behavioral engagement	0.871	0.878	0.897	0.508
Cognitive engagement	0.927	0.931	0.938	0.561
Emotional engagement	0.827	0.862	0.760	0.587

**Table 8:** Discriminant validity.

**Matrix**	**Attitude toward structure feedback**	**Behavioral engagement**	**Cognitive engagement**	**Emotional engagement**
Attitude toward structure feedback	0.811			
Behavioral engagement	0.806	0.713		
Cognitive engagement	0.791	0.651	0.749	
Emotional engagement	0.705	0.687	0.652	0.766

In testing the validity of the model constructs, two measures were considered, which are convergent validity and discriminant validity, where convergent validity was employed to assess whether items within the same construct were highly correlated with each other. Moreover, discriminant validity was used to assess if the items loaded more on their intended construct than on other constructs (Lai and Chen, 2011). Therefore, construct validity was tested using factor analysis with principal component analysis and varimax rotation. The diagonal line of loading between 0.45 and 0.54 is generally considered fair, loading between 0.55 and 0.62 is good, loading between 0.63 and 0.70 is very good, and loading is considered excellent if it is higher than 0.71 (Comrey and Lee, 2013). The modified factor loading analysis indicated that all the constructs in the model have both excellent convergent and discriminant validity with each AVE value greater than the threshold value, as shown in Table 8.

According to Table 9, the factors used in this study show significantly supported remarks regarding the PLS-SEM grogram. Therefore, this model shows a high correlation between these factors.

**Table 9:** Bootstrapping path coefficient.

	**Original sample (O)**	**Sample mean (M)**	**Standard deviation (STDV)**	***T* statistics (O/STDV)**	***P*-value**	**Remarks**
Attitude toward structure feedback → behavioral engagement	0.806	0.808	0.050	16.088	0.000	Supported
Attitude toward structure feedback → cognitive engagement	0.791	0.793	0.056	14.004	0.000	Supported
Attitude toward structure feedback → emotional engagement	0.705	0.713	0.045	15.829	0.000	Supported

## Discussion

Teaching may be extremely rewarding when students are engaged, profoundly interested in the subject matter, and intelligently participating. However, strong student engagement is difficult to create. This study promotes structured feedback *via* online education as one of these ways since the ability to adapt and nurture improved student engagement frequently involves research and preparation. Student engagement is crucial in every class, but it is essential in the online learning environment where students must be disciplined enough to avoid distractions and other obligations competing for their time while being cut off from their instructor and other students. According to extensive studies in class engagement, student's engagement differs depending on the environment that is established by the school and instructor as well as the learning opportunities that are provided in the classrooms, which is in notable agreement with this study, as the environment turned to online one (Watanabe, 2008; Kelly and Turner, 2009; Nasir et al., 2011).

According to the results of this study, all three types of engagement (cognitive, behavioral, and emotional) are positively correlated with the structured feedback used in online classes, which is in line with the following studies that used different types of feedback in online classes (Flahery and Pearce, 1998; Dixson, 2010; Chakraborty and Nafukho, 2014; Martin and Bolliger, 2018). So, the three types of engagement were achieved during class time, which is in agreement with Latipah et al. (2020). The type of structured feedback that is used in this study led the students to ask themselves what I know, what I want to know, and what I learned, in each lesson, which shows positive to very positive results in relation to students' engagement. Wenger (1998) and Vonderwell and Zachariah (2005) stated that participating in an online class involves more than just joining the class or commenting on a message board. These researchers concluded that participating in a discussion and being active are crucial components of being engaged.

Although students can post to the discussion board, real engagement occurs in the dialogue that develops after the first post. The environment of online classes that make learners passive receivers of knowledge, just listeners, specifically in human science lectures, requires a rethinking process of the ways used in presenting the material, which is in agreement with Garrison et al. (2000) who stated that “potential for creating an educational community” referring to the exploitation of subject material. Hence, teachers need to activate the ideas of student-centered education with some types of class discussion (Mandernach et al., 2006) to ensure wide participation of the students in the class, which in turn develops their class engagement. The results of the study in relation to each question can be stated as follows:

### First research question

#### Is there a correlation between EFL college students' cognitive engagement and their attitudes toward structured feedback in online education?

Depending on the results gained, cognitive engagement achieved very positive results in relation to structured feedback between the two others. This result demonstrates that leading students to think in an online class can play a role in increasing their engagement, specifically cognitive engagement (Nguyen et al., 2016; Pilotti et al., 2017). Let us first clarify cognitive psychology and discuss how it affects student engagement and active learning. According to cognitive psychology, active learning entails the growth of cognition, which is accomplished through accumulating systematic knowledge structures and methods for understanding, remembering, and solving issues, and these processes are related to applying structured feedback. Active learning also involves an interpretation process, whereby new information is connected to previously learned information and retained in a way that emphasizes the extended significance of these links, and this can interpret the positive results in relation to cognitive engagement in the current study.

At the start of a new unit or lesson, online teachers routinely provide context and meaning to students, which promotes improved retention and mastery. Cognitively speaking, because memory is associative, the environment can influence the information and vice versa. When new memories are generated, neurons wire together. Students' curiosity and learning capacity might be piqued by a teaching technique that uses questions to guide lesson ideas, and this explanation can provide another justification for the positive results of cognitive engagement in relation to structured feedback in online education.

The results of the present study are in agreement with Mandernach (2009) who claims that an online course encourages the best level of student cognitive involvement if it:

- Incorporates authentic learning tasks and active learning environments.
- Encourages personal connections between students and teachers in the class.
- Helps to learn to take place in a virtual setting.

It is evident that all these three points mentioned by Mandernach (2009) exist in the current study, so for this reason, the results are notably positive in relation to the existence of cognitive engagement in the online environment.

### Second research question

#### Is there a correlation between EFL college students' behavioral engagement and their attitudes toward structured feedback in online education?

In relation to the results achieved, behavioral engagement is positively correlated with structured feedback in online education. Results prove that if the students interact behaviorally in class, this will affect positively their behavioral engagement and this result is in line with Nguyen et al. (2016). The behavioral engagement domain asks about how students behave in class, how they participate in extracurricular activities, and how interested they are in their academic assignments. All these three domains are under the light in the present study as they are all related to how EFL college students engage behaviorally *via* online education using structured feedback. The varied activities used in structured feedback lead the students to ask; what I know, what I want to know, and what I learned and try to provide answers for all of them, encouraging the students to engage indirectly with their class activities, and show interest in applying them.

Focusing on the student's support during the activities (such as attendance and pleasant interactions), research on school participation has shed light on the student's motivation to participate in school to gain positive class engagement (Jones et al., 2008; Wang and Holcombe, 2010). So, it is clear that the right choice of class activity plays a crucial role in activating students' engagement as a result of raising their motivation. The students' interest in their academic assignment, which refers to the concrete behavioral acts displayed by the students to demonstrate their desire to participate in classroom activities and their will to tackle difficult material, is also a pivotal component of behavioral engagement. Research in this area sheds light on the classroom exercises that result in the student displaying concrete behavioral engagement, such as perseverance, concentration, asking questions, and participating in different class discussions (Yazzie-Mintz and McCormick, 2012; Cooper, 2014).

Engaging students as autonomous learners in online contexts without the presence of a teacher is still complex. This has prompted more research to be done on the variables affecting students' engagement in this situation. Student time-on-task behavior is referred to engagement with the assigned activities. Throughout the class, students were required to participate in a variety of activities, specifically in online education to prove their active existence in class and to be engaged gradually.

Another point is where the students showed strong engagement in the feedback on various activities. As in the present study, it is discovered that this feature was excellent for learning, specifically online learning. For instance, after receiving feedback that revealed their presumptive understanding to be false, students revisited the simulation model and further investigated the concepts. It helped them to better comprehend what was going on at the molecular level. This research can generally be divided into three primary categories: interactions between students and the teacher, interactions between students and their peers, and interactions between students and the subject matter. All these categories are part of the structured feedback used in the current study. which proves its effectiveness in activating students' engagement of all types.

### Third research question

#### Is there a correlation between EFL college students' emotional engagement and their attitudes toward structured feedback in online education?

According to the results, emotional intelligence was correlated positively with using structured feedback in online education. Due to the nature of online learning as a distant learning experience, there are obstacles to student engagement and learning. It is observed that low student engagement and greater dropout rates in online courses are primarily a result of these impediments. Emotions are a potent weapon in the struggle for online student attention, engagement, and persistence (Deng, 2021). The difficulties of forming social and emotional connections with and between them are the biggest obstacle to the success of online students (Gallien and Oomen-Early, 2008).

However, combining technology and emotion in the classroom might have an even greater impact. Creating an emotional connection to the material inside the classroom can be a vital tool for student retention. Students will be better able to comprehend, relate, and recognize the significance of the course subject through powerful teaching techniques, such as structured feedback. They will not perceive the course material as meaningless facts; instead, it might elicit an emotional reaction that makes it easier for them to retain it for the duration of the course (Wang et al., 2022a,b; Wang J. et al., 2022). Hence, the role of the teacher in nurturing emotional intelligence in an online class is crucial and essential (Al-Obaydi et al., 2022).

Online classes can also be used to address students' emotions. Discussing hot-button issues that concern students is one way to do this. Designing discussion platforms, like structured feedback, such that students can express their own emotions and that automatically encourage engagement (Deng, 2021). This viewpoint is also expressed by Niedenthal et al. (2006) who mentioned that learning is cognitive and emotional simultaneously. To ensure that students treat one another with respect despite any disputes, it is crucial to set ground rules before these dialogues. Overall, rather than having students study content inside a framework of monotony, it is critical to use technology to evoke emotion and keep content exciting.

Online social and emotional support for students will boost their engagement, perseverance, learning, and success. Helping students get through the obstacles that arise because of the distance involved with taking online classes is, in fact, one of the best things online instructors can do (Xie and Derakhshan, 2021). In this concern, (Rodríguez-Ardura and Meseguer-Artola, 2016, p.100) stated that many learners “feel individually placed within a true humanized education environment” in which they feel that they are taking part “in a true teaching-learning process, interacting with their lecturers and peer students.” Finally, the more alternatives we can give to online students in online classes, the more they engaged in all three types of engagement and the more control they will have over their education, and the ability to choose activities that are important to their personal, academic, and professional goals.

## Conclusion

The focus on the students and how tactics in online education classes affect their participation and learning in the classroom from their point of view is what makes this study significant. The use of structured feedback in an online class for EFL college students, which gave the students a chance to think, speak, participate, express, and compete at the same time proves its successful utilization in a college context. For the following reasons, this study will be necessary for higher education institutions and professors instructing distance learning programs. The results of this study may help administrators and teachers in online education make judgments on engagement tactics for current online education courses. Numerous prior studies on online education of all types have been conducted from the perspective of the faculty; this study would enable the faculty to learn about successful engagement techniques by hearing from the students.

In addition, it is clear that improved learner engagement does not always result from the use of online platforms. When using online education, it is essential to carefully plan educational tactics to boost and maintain learner engagement. As a drawback, giving this type of feedback may have the unintended consequence of decreasing student learning autonomy. Making the student an independent learner is the major goal of the online education environment. Therefore, when proper teaching methods are offered in this sort of learning, a balance between individualized learning and reducing the teacher's involvement is always preferred. Thus, it has been suggested that more work can be done researching the negative and positive sides of using feedback in online education.

## Data availability statement

The original contributions presented in the study are included in the article/Supplementary material, further inquiries can be directed to the corresponding author.

## Ethics statement

The studies involving human participants were reviewed and approved by Golestan University. The patients/participants provided their written informed consent to participate in this study.

## Author contributions

All authors listed have made a substantial, direct, and intellectual contribution to the work and approved it for publication.
