# Supplementary material for: What I know, what I want to know, what I learned: Activating EFL college students' cognitive, behavioral, and emotional engagement through structured feedback in an online environment
Source: Front Psychol. 2023 Jan 4;13:1083673. doi: 10.3389/fpsyg.2022.1083673 (PMC9845881; doi:10.3389/fpsyg.2022.1083673)
Supplement: Supplementary file 1 [file Data_Sheet_1.docx]

**Appendix 1**

**1. Student Engagement in Schools Questionnaire (SESQ)**

**1.1. Affective**

I am very interested in learning online.

I think what we are learning in online class is interesting.

I like what I am learning in online class.

I enjoy learning new things in online class.

I think learning in online class is boring.

I like my college.

I am proud to be at this college.

I look forward to going to college.

**1.2. Behavioral**

I try hard to do well in my online class.

When I’m in online class, I participate in class activities.

I pay attention in online class.

I just act like I’m working.

In online class, I do just enough to get by.

When I’m in online class, my mind wanders.

If I have trouble understanding a problem, I go over it again until I understand it.

When I run into a difficult homework problem, I keep working at it until I think I’ve solved it.

I am an active participant of in online activities.

I volunteer to help with online class activities.

I take an active role in extracurricular activities in my online class.

**1.3. Cognitive**

When I’m in online class, I try to understand the material better by relating it to things I already know.

When I’m in online class, I figure out how the information might be useful in the real world.

When learning new information, I try to put the ideas in my own words.

When I’m in online class, I try to connect what I am learning with my own experiences.

I make up my own examples to help me understand the important concepts I learn from my online class.

When learning things from my online class, I try to see how they fit together with other things I already know.

When learning things from my online class, I often try to associate them with what I learnt in other classes about the same or similar things.

I try to see the similarities and differences between things I am learning for class and things I know already.

I try to understand how the things I learn in online class fit together with each other.

I try to match what I already know with things I am trying to learn for online class.

I try to think through topics and decide what I’m supposed to learn from them, rather than studying topics by just reading them over.

When studying, I try to combine different pieces of information from course material in new ways.

**2. Students’ attitudes towards structure feedback**

I learn interesting things in online “method of teaching” lesson.

“Method of teaching” lesson is exciting.

“Method of teaching” is one of my best subjects.

We learn “Method of teaching” better when using structured feedback.

In my “Method of teaching” , I understand everything.

I like the activities in this class like giving feedback.

I think it is good to give your feedback toward the material.

Feedback in class is important.

The benefits of feedback are greater than the harmful effects in online “method of teaching”.

There are many exciting things happening in online “method of teaching”.
